# Clinical applicability of and changes in perfusion MR imaging in brain metastases after stereotactic radiotherapy

**DOI:** 10.1007/s11060-018-2779-7

**Published:** 2018-02-01

**Authors:** M. Kerkhof, I. Ganeff, R. G. J. Wiggenraad, G. J. Lycklama à Nijeholt, S. Hammer, M. J. B. Taphoorn, L. Dirven, M. J. Vos

**Affiliations:** 1 Department of Neurology, Haaglanden Medical Center, PO Box 432, 2501 CK The Hague, The Netherlands; 2 Department of Radiotherapy, Haaglanden Medical Center, The Hague, The Netherlands; 3 Department of Radiology, Haaglanden Medical Center, The Hague, The Netherlands; 4 Department of Neurology, Leiden University Medical Center, Leiden, The Netherlands

**Keywords:** Perfusion MR imaging, Brain metastases, Stereotactic radiotherapy

## Abstract

To assess the applicability of perfusion-weighted (PWI) magnetic resonance (MR) imaging in clinical practice, as well as to evaluate the changes in PWI in brain metastases before and after stereotactic radiotherapy (SRT), and to correlate these changes to tumor status on conventional MR imaging. Serial MR images at baseline and at least 3 and 6 months after SRT were retrospectively evaluated. Size of metastases and the relative cerebral blood volume (rCBV), assessed with subjective visual inspection in the contrast enhanced area, were evaluated at each time point. Tumor behavior of metastases was categorized into four groups based on predefined changes on MRI during follow-up, or on histologically confirmed diagnosis; progressive disease (PD), pseudoprogression (PsPD), non-progressive disease (non-PD) and progression unspecified (PU). Twenty-six patients with 42 metastases were included. Fifteen percent (26/168) of all PW images could not be evaluated due to localization near large vessels or the scalp, presence of hemorrhage artefacts, and in 31% (52/168) due to unmeasurable residual metastases. The most common pattern (52%, 13/25 metastases) showed a high rCBV at baseline and low rCBV during follow-up, occurring in metastases with non-PD (23%, 3/13), PsPD (38%, 5/13) and PU (38%, 5/13). Including only metastases with a definite outcome generally showed low rCBV in PsPD or non-PD, and high rCBV in PD. Although non-PD and PsPD may be distinguished from PD after SRT using the PW images, the large proportion of images that could not be assessed due to artefacts and size severely hampers value of PWI in predicting tumor response after SRT.

## Introduction

About 10–30% of patients with systemic cancer develop brain metastases. The overall median survival in 3940 patients with newly diagnosed brain metastases was 7.2 (range 2.8–25.3) months depending on tumor type, number of brain metastases, presence of extracranial metastases and patient-related factors such as age and performance status [1]. Treatment may involve resection, radiotherapy (stereotactic techniques or whole brain radiotherapy), systemic treatment or a combination of these. Radiotherapy may result in adverse radiation effects (ARE) comprising a spectrum of radiation effects with (temporary) enlargement of the area of contrast-enhancement in tumor and surrounding normal brain tissue, which may be reversible or irreversible [2]. The term pseudoprogression is used when there is an early delayed injury and when this is a reversible reaction. The other end of the spectrum is radiation necrosis, which is an irreversible reaction and late complication of radiation to the brain [3]. In literature, these terms are used interchangeably, but in this study the AREs are referred to as pseudoprogression. MR imaging for follow-up after radiotherapy may either show stable or a decreased area of contrast enhancement (i.e., non-progressive disease; non-PD), or increased contrast enhancement [i.e., progressive disease (PD) or pseudoprogression (PsPD)]. However, the distinction between PD and PsPD cannot be made easily with conventional MR imaging. Several advanced imaging methods based on MRI, such as delayed-contrast MRI to calculate treatment response assessment maps (TRAMs), proton magnetic resonance spectroscopy (MRS), and positron-emission-tomography (PET) are studied in patients with brain metastases [4–8]. With the TRAMs approach, to differentiate tumor from nontumor tissue, a sensitivity and positive predictive value of 100% respectively 89% was found in patients with brain metastases [7]. To distinguish between PD and PSPD MRS demonstrated to have sensitivity between 33 and 50% and a specificity of 100% [8]. Another advanced technique is perfusion MR imaging which may provide additional information necessary to make the distinction between PD and PSPD. The capability of perfusion-weighted imaging (PWI) to differentiate tumor recurrence from PsPD of cerebral metastases after radiotherapy has been described before in four studies evaluating predictive value of PWI in brain metastases treated with radiotherapy [9–12]. For predicting tumor recurrence, visual inspection of the relative cerebral blood volume (rCBV) map yielded a sensitivity and specificity of 70 and 93%, respectively [9], while quantitative PWI analysis resulted in a sensitivity between 70 and 91% and a specificity between 73 and 100% [9, 10]. Moreover, a decrease in rCBV of > 15% six weeks after radiotherapy was found to be predictive of tumor response after six months, with a sensitivity of 91% and specificity of 71% [11]. Similarly, a decreased rCBV after 1 week of treatment with SRT or WBRT (*p* < 0.05) was found to be predictive of tumor response 1 year post-treatment at last available follow-up [12]. Interestingly, in this study a reduction of rCBV after one month was also seen in patients with PD (sensitivity 74%, specificity 82%). Although the previous studies found that perfusion MRI is a useful tool in the distinction of PD and PsPD, these studies only described the changes of perfusion MR parameters in patients with radiological progression. However, it is currently unknown if these patterns are unique for patients with progression and do not occur in patients with radiologically stable lesions. In order to get better insight in the effect of radiotherapy on perfusion MRI parameters, we also included patients with radiologically stable disease in our study. The aim of this study was therefore to evaluate the applicability of the PW imaging technique and changes in PWI in brain metastasis after SRT and to study the rCBV patterns in relation to changes in the area of contrast-enhancement.

## Patients and methods

### Patient population

We retrospectively studied patients with one to three brain metastases who received SRT between January 2011 and December 2013 at the Radiotherapy Center West in The Hague, The Netherlands. Only patients with baseline conventional and perfusion MR and at least at 3 and 6 months follow-up were included. Patients with prior resection or radiotherapy, and patients who received subsequent (whole brain) radiotherapy within 6 months post-SRT, were excluded. Recorded demographic and clinical parameters included age, gender, date of birth, age at diagnosis, diagnosis and location of primary tumor, date of diagnosis, date of first SRT and metastases location.

### Radiation therapy

Patients were treated with Dynamic Arc Technique. Prescribed doses, specified on the 80% isodose, were 1 × 18, 1 × 21, 3 × 8 Gy or 3 × 8.5 Gy, depending on the volume of the planning target volume (PTV). A CTV (clinical target volume)-PTV margin was given to all patients. Patients received dexamethasone (6 mg twice a day) from the day before SRT until 1 day after SRT. Depending on previous use, dexamethasone was either stopped or tapered based on symptoms.

### Magnetic Resonance Imaging

MR imaging of the brain was performed (1.5 T, Siemens Avanto, Siemens Medical Solutions, Erlangen, Germany) according to the brain tumor protocol of the hospital. Imaging included T1-weighted (T1WI) pre- and post-contrast images, T2-weighted images (T2WI) and PWI. PWI was acquired using a gradient echo echoplaner sequence (GE-EPI). Slice thickness of T1WI is 1.3 mm. A contrast prebolus 0,1 ml/kg gadolinium followed by 10 cc NaCl (2 cc/s) was given to correct for contrast leakage. PW images were obtained during the first pass of gadolinium (20 cc, 4 cc/s) with an injection delay of 10 s. Imaging parameters of PWI were: repetition time/echo time (TR/TE) 1490/30 ms, slice thickness 5.0 mm, field of view 230, acquisition matrix 128/128, flip angle 90°. MR imaging was performed at baseline, 3 and 6 months after SRT. MR images were anonymized before evaluation.

### Assessment of MR images: lesion size

Baseline and standard follow-up metastatic size at 3, 6 and when available 9 and 12 months after SRT were evaluated. Measurements of the estimated area of contrast-enhancement were obtained from axial post-contrast T1-weighted images by selecting the largest tumor diameter and the greatest perpendicular diameter [13, 14]. The tumor responses of metastases were categorized into four groups based on changes in contrast enhancement on T1-weighted images during follow-up or based on a histologically confirmed diagnosis; (1) progressive disease (PD), (2) pseudoprogression (PsPD), (3) non-progressive disease (non-PD) and (4) progression unspecified (PU).All metastases showing a decrease of at least 5% (to ascertain a true change in tumor size, whether or not clinically relevant) in tumor size over time were categorized as non-PD. Metastases with an initial increase in size (≥ 5%), but without a subsequent decrease in size (≥ 5%), were categorized as PU. This group may include both PsPD and PD, which could not be further specified based on (missing) histology or follow-up. PsPD was defined as a decrease of size on T1WI after an initial increase of contrast enhancement of at least 5%. Definite PD was based on a histological diagnosis consisting of viable tumor tissue.

### Assessment of MR images: PW imaging

rCBV was assessed by subjective visual inspection of the rCBV maps in the contrast-enhanced area. This visual score was based on presence or absence of highly vascularized areas within the contrast-enhanced lesion relative to the contralateral hemisphere and was defined as high rCBV versus low rCBV, reflecting viable tumor tissue or treatment-related effects, respectively, or as not assessable. The cut-off used to define a metastasis as unmeasurable was < 60 mm^2^.

All MR assessments were performed independently by two experienced neuroradiologists (GL, BH). Parameters included in the evaluation were the quality of the scan, T1-assessment of contrast enhancement, and PW images results. Discordant results on the scoring form between the two radiologists were resolved by consensus. For PWI pattern analysis, a minimal of two PWI follow-up time points (3 and 6 months) were necessary. PWI patterns for perfusion changes in relation to the estimated area of contrast-enhancement weres studied for all four categories. Additionally, we performed a PWI subanalysis in which we only included those metastases with a definite outcome.

### Statistical analysis

Descriptive statistics were used to define the patient population. Survival time was calculated from the first day of SRT until the date of death or the last date of follow-up when the patient was still alive. Descriptive statistics were also used to study the rCBV patterns for perfusion changes in relation to the estimated area of contrast-enhancement during follow-up. Statistical analysis was performed using SPSS version 23 (SPSS, Chicago, IL). Differences between categorical factors were assessed by the Chi-Squared test (χ2) or Fisher’s Exact test. All tests were two-tailed, and *P* < 0.05 was considered to be statistically significant.

## Results

### Patient selection, clinical outcome and survival

A total of 133 patients with 224 metastases were treated with SRT between 2011 and 2013. Of these, 26 patients with 42 metastases were eligible according to our inclusion criteria (Table 1). More than half of the patients were female (54%) and the median age was 66 years (range 40–84 years). Primary cancer sites included non-small cell lung carcinoma (NSCLC) (46%), breast cancer (19%) and others (36%) (melanoma, gastro-intestinal cancer and urogenital cancer). The median survival time was 17 (range 10–22) months. After 1 year of follow-up, four patients (15%) were still alive. Eleven out of 26 patients (42%) had multiple metastases. The dosage of radiation varied from 18 to 25,5 Gy depending on metastasis size and location, with a median of 21 Gy.

**Table 1:** Patient characteristics

Gender, n (%)	
Male	12 (46)
Female	14 (54)
Dosage SRT (Gy)	
Median	21
Range	21–24
Age (years)	
Median	65
Range	40–84
Primary tumor, n (%)	26
NSCLC	12 (46)
Breast cancer	5 (19)
Other	9 (36)
Metastasis per primary tumor, n (%)	42
NSCLC	20 (48)
Breast cancer	9 (21)
Other	13 (31)
Amount of metastases, n (%)	
1	15 (58)
2	6 (23)
3	5 (19)
Metastasis location, n (%)	
Supra-tentorial	36 (86)
Infra-tentorial	6 (14)

*n* number, *NSCLC* non-small cell lung carcinoma

### MR lesion size

Changes in the estimated area of contrast-enhancement on T1WI with gadolinium were evaluated on a group level after 3 (n = 42), 6 (n = 42), 9 (n = 25) and 12 (n = 16) months follow-up (Table 2).

**Table 2:** Changes in the estimated area of contrast-enhancement on T1WI with gadolinium, compared to the previous time point

Changes T1WI with gadolinium	3 months FU (N = 42) (%)	6 months FU (N = 42) (%)	9 months FU (N = 25) (%)	12 months FU (N = 16) (%)
Decrease	33 (79)	24 (57)	9 (36)	10 (63)
Increase	9 (21)	17 (41)	14 (56)	4 (25)
Stable	–	1 (2)	2 (8)	1 (6)

An increase is defined as ≥ 5% increase in the area of contrast-enhancement compared to previous time point and a decrease as ≥ 5% decrease in the area of contrast-enhancement compared to the previous time point

The median metastases size before SRT was 290 mm^2^ (range 77–591 mm^2^). Median size 3 and 6 months post-SRT was 86 mm^2^ (30–356 mm^2^) and 149 mm^2^ (range 12–500 mm^2^) respectively. Three months after SRT, 79% (33/42) of the metastases decreased in size or remained stable in size compared to the size at baseline, whereas 6 months after irradiation only 60% (25/42) showed a decrease in size or had a stable size compared to the size at 3 months. After 9 and 12 months follow-up, 44% (11/25) and 69% (11/16) showed a decrease in size or remained stable compared to the size at 6 and 9 months follow-up, respectively. From baseline until 6 months after radiotherapy, 26% (11/42) of the metastases showed an increased area of contrast-enhancement and 74% (31/42) showed a decrease in size on T1WI with gadolinium.

At the end of follow-up, 18 out of 42 metastases were classified as PU (43%), 15 as non-PD (36%), eight as PsPD (19%) and one metastasis as PD (2%).

### MR PW imaging

**Fig. 1:**
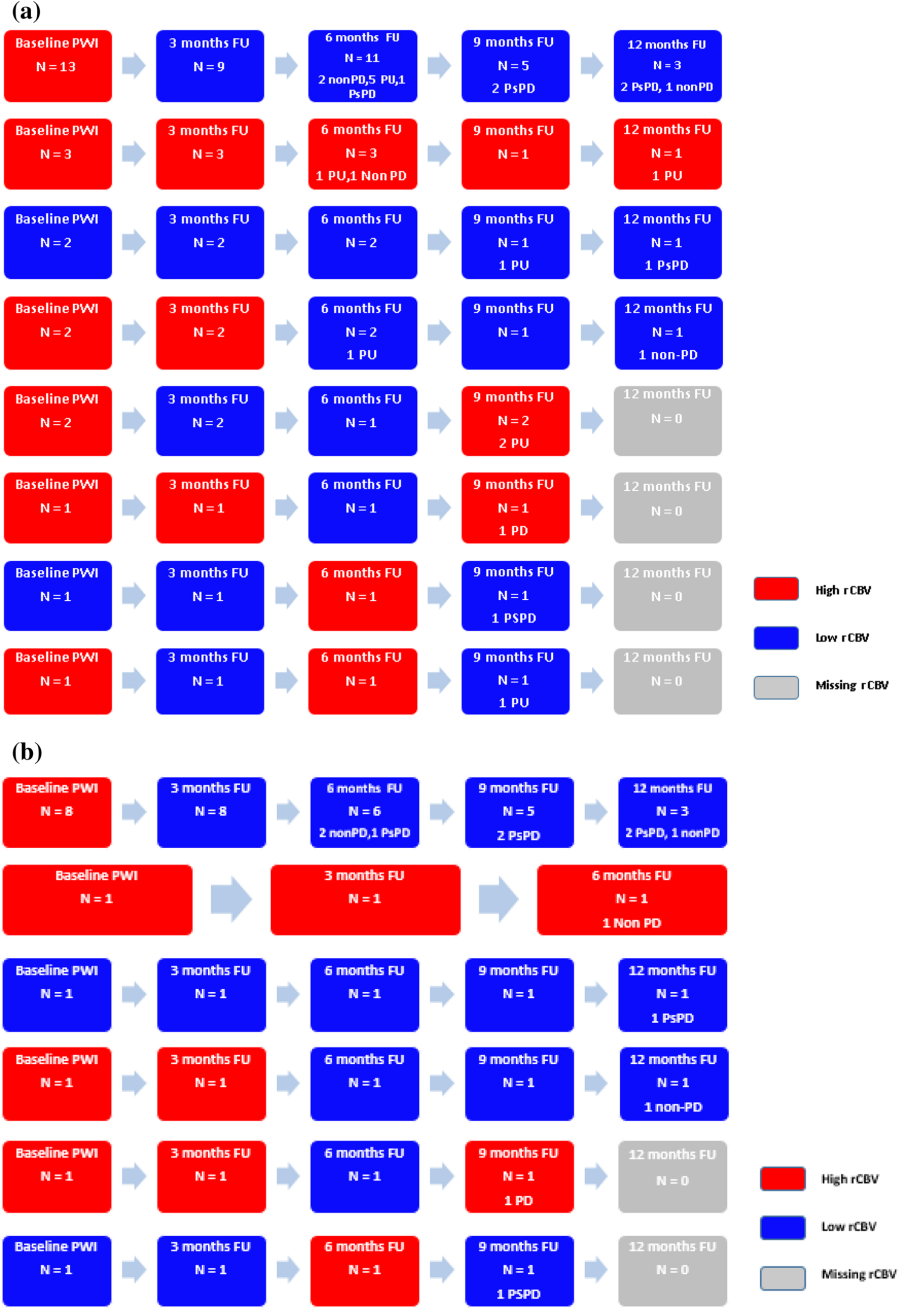
**a** PWI pattern analysis (N = 25) and **b** PWI subanalysis (N = 13) showing high or low rCBV at baseline and during follow-up. Numbers of metastases evaluated at each time point are added as well as the number of patients with PsPD, non-PD, PU and PD in case it is their last follow-up moment. *Red colour* high rCBV, *blue colour *  low rCBV, *PWI* perfusion weighted image, *FU* follow-up, *PsPD* pseudoprogression, *non-PD* no progressive disease, *PU* progression unspecified (progressive disease or pseudoprogression), *PD* progressive disease

A total of 168 PW images at baseline and follow-up were reviewed. Up to forty-six percent (78/168) of all PW images could not be used for PWI analysis; thirty-one percent (52/168) could not be evaluated due to unmeasurable residual metastases (< 60 mm^2^), while the other fifteen percent (26/168) could not be evaluated due to localization near large vessels or the scalp (n = 13), or due to the presence of hemorrhage artefacts (n = 13). The lesions which could not be used were not included in further PWI results. Thirty-two metastases (76%, 32/42) remained for baseline PWI analyses and twenty-five metastases (60%, 25/42) remained for follow-up PWI pattern analyses with a minimum of two PWI follow-up time points (3 and 6 months); 21/42 (50%) PWI analyses at 3 months and 22/42 (52%) at 6 months follow-up. At 9 and 12 months follow-up, only 13/42 (31%) and 6/42 (14%) PWI analyses were available. No association was found between primary tumor type and rCBV 3 and 6 months after irradiation (*p* = 0.484 and *p* = 0.940, respectively). Of the metastases suitable for analysis at baseline, 84% (27/32) showed high rCBV. Three months post-SRT, only 29% (6/21) showed high rCBV. At 6, 9 and 12 months, 23% (5/22), 31% (4/13) and 17% (1/6) showed high rCBV, respectively. For each metastasis, we have also evaluated the individual pattern of rCBV flow (Fig. 1a). After radiotherapy, the most frequent pattern (52%, 13/25 metastases) showed a high rCBV at baseline and low rCBV during follow-up. However, this pattern was independent of the subsequent tumor status category: 3/13 (23%) were subsequently categorized as non-PD, 5/13 (38%) as PsPD and 5/13 (38%) as PU. The other metastases did not fit into any pattern and were not related to specific categories based on the change of contrast-enhancement. Of the seven metastases in the PWI analyses categorized as PsPD, six (86%) had a continuously low rCBV during follow-up. Of the five metastases categorized as non-PD, three showed a continuously low rCBV during follow-up (60%). The patient with histologically confirmed PD was found to have a low rCBV at 6 months and a high rCBV at 3 and 9 months of follow-up.

The subanalysis contained only the metastases with a definite outcome; PsPD, PD and non-PD. A total of 13 metastases were included. In 12/13 (92%) the follow-up PWI demonstrated a concordant result with the changes in the estimated area of contrast enhancement; low rCBV in case of PSPD or non-PD and high rCBV in case of PD (Fig. 1b). However, one metastasis categorized as non-PD demonstrated a high rCBV at baseline and at 3 and 6 months of follow-up.

## Discussion

The differentiation between PsPD and PD in patients with brain metastases after SRT may have clinical implications. If PD could be diagnosed reliably, patients can receive timely and appropriate additional anti-tumor treatment, whereas patients with PsPD should not be treated in the same way. Although some authors have suggested that all lesions increasing in size resulting in neurological problems should be treated, it is a matter of debate whether this should be done with anti-tumor or supportive treatment. The use of quantitative perfusion MRI for this indication showed promising results, but evidence for the widely used visual technique in clinical practice for PWI interpretation is limited [9–13]. In addition, previous studies investigating the use of quantitative perfusion MRI only described the changes of perfusion MR parameters in patients with radiological progression, limiting generalizability of these findings.

In the current study we described the change of the estimated area of contrast-enhancement in brain metastases from baseline up to a minimum of 6 months after irradiation. This selection criterion impacted the overall survival of this study population, which is high compared to the median survival of a general brain metastases patient population. Six months after radiotherapy most of the metastases in our study initially decreased in size compared to baseline (74%). Based on the change of the area of contrast-enhancement over time (and when available based on histology) metastases were categorized as PD, PsPD, non-PD or PU. Contrary to other studies on this subject, the lesions that increased over time without histological confirmation or a subsequent decrease of contrast-enhancement were categorized as the unspecified (PU) cases. To eliminate the risk of false classification, we chose to not further specify the tumor status [9, 10].

In the literature, an occurrence of 20% PsPD was described in glioma patients treated with temozolomide chemo-radiation [14]. Of the brain metastases patients with progressive contrast enhancement during follow-up, 25–41% were classified as PsPD [9, 10, 15, 16]. Diagnoses were based on histology, definite radiological decrease or a combination of radiological and clinical follow-up. We found a significant reduction in the area of contrast-enhancement in 79% of the metastases 3 months after SRT. However, 6 months after irradiation, in a large number of metastases (41%) the area of contrast-enhancement increased again due to either PD or PsPD. In the clinical setting this can be a difficult moment in decision-making. Most studies attempting to make the distinction between these two entities, describe only 3 months of follow-up [9–11]. However, we demonstrated that 41% of lesions do increase after this follow-up interval.

The strength of this study is that all patients, independent of tumor status, were included, whereas most studies on perfusion imaging included only patients with radiological progression. Sixty percent of the metastases categorized as non-PD had a continuously low rCBV during follow-up.

### Study limitations

Unfortunately, almost half of the metastases (43%) in our study were categorized as PU, making drawing conclusions hardly possible. This limitation is partly due to the lack of histology in almost all patients. On the other hand, this reflects clinical practice, in which treatment choices have to be made on the limited available evidence.

Furthermore, PWI were assessed using the visual method, which is a subjective method widely used in clinical practice. Although widely used, large interobserver variability, observed in evaluating rCBV in patients with glioblastoma, questions the value of this method [13]. Moreover, the perfusion MRI was not applicable in several metastases; in almost half of the PW images the rCBV could not be determined due to small lesion size or artefacts, which is a major limitation. Artefacts in PWI may be based on localization of the metastases near large vessels, localization in the posterior cranial fossa, necrosis or hemosiderin deposition. The latest is thought to be due to small haemorrhages in the tumor bed, caused by radiation therapy [17]. A hemosiderin rim could indicate radiation-induced damage to the metastases. The rCBV may not be reliable when bleeding has occurred, because bleeding within the tumor could cause false increase or decrease in rCBV [18]. Therefore, caution in interpretation is warranted in case of haemorrhage. After excluding metastases with impeding artefacts and metastases too small for assessment, only 22 patients with 25 metastases remained available for further PWI analysis. The sample size is an important limitation of the study and limited the interpretation of the study results.

## Conclusion

Most metastases showed a decrease in the area of contrast-enhancement 3 months after irradiation, reflecting the known efficacy of SRT. The follow-up MRIs learnt us more about rCBV development after SRT. The majority of brain metastasis (52%) had a high baseline and low follow-up rCBV, independent of the eventual tumor status: low perfusion during follow-up is seen in patients with both PD, non-PD and PsPD. Based on these results it can be concluded that the visual method of PWI analysis does not provide unequivocal guidance in predicting progression of metastasis. However, when excluding metastases that were classified as having PU from the analysis, results of the PWI subanalysis were concordant with the changes in the area of contrast-enhancement in almost all patients, with low rCBV in case of PSPD or non-PD and high rCBV in case of PD in 12 out of 13 patients. This suggests that non-PD and PsPD may be distinguished from PD based on the visual method of the PWI analysis. Nevertheless, the large proportion of PW images that could not be assessed due to artefacts and size severely hampers the ability to predict tumor response.
